# High-Density Mapping of an Adult-Plant Stripe Rust Resistance Gene *YrBai* in Wheat Landrace Baidatou Using the Whole Genome DArTseq and SNP Analysis

**DOI:** 10.3389/fpls.2018.01120

**Published:** 2018-08-02

**Authors:** Qiang Li, Juan Guo, Kaixiang Chao, Jinye Yang, Weiyun Yue, Dongfang Ma, Baotong Wang

**Affiliations:** ^1^State Key Laboratory of Crop Stress Biology for Arid Areas, College of Plant Protection, Northwest A&F University, Yangling, China; ^2^Tianshui Institute of Agricultural Sciences, Tianshui, China; ^3^College of Agriculture, Yangtze University, Jingzhou, China

**Keywords:** *Puccinia striiformis* f. sp. *tritici*, adult-plant resistance, wheat landrace, molecular mapping, DArTseq

## Abstract

Stripe rust, caused by the biotrophic fungus *Puccinia striiformis* f. sp. *tritici* (*Pst*), is one of the most widespread and destructive wheat diseases worldwide. Growing resistant cultivars is an effective approach for controlling this disease. However, because host resistance genes were easily overcome by new virulent *Pst* races, there is a continuous demand for identifying new effective wheat stripe rust resistance genes and develop closely linked markers for marker-assisted selection (MAS). Baidatou, an old Chinese wheat landrace, has been grown for several decades in Longnan region, Gansu Province, where stripe rust epidemics are frequent and severe. In our previous study, a single dominant gene *YrBai* in Baidatou was identified to control the adult-plant resistance (APR) to Chinese prevalent *Pst* race CYR33. And the gene was located on wheat chromosome 6DS by four polymorphic simple sequence repeat (SSR) and two sequence-related amplified polymorphism (SRAP) markers, with the genetic distances of two closely linked markers 3.6 and 5.4 cM, respectively. To further confirm the APR gene in Baidatou and construct the high-density map for the resistance gene, adult plants of F_1_, F_2_, F_3_, and F_5:6_ populations derived from the cross Mingxian169/Baidatou and two parents were inoculated with CYR33 at Yangling field, Shaanxi Province during 2014–2015, 2015–2016, and 2016–2017 crop seasons, respectively. The field evaluation results indicated that a single dominant gene confers the APR to *Pst* race CYR33 in Baidatou. 92 F_3_ lines and parents were sequenced using DArTseq technology based on wheat GBS1.0 platform, and 31 genetic maps consisted of 2,131 polymorphic SilicoDArT and 952 SNP markers spanning 4,293.94 cM were constructed. Using polymorphic SilicoDArT, SNP markers and infection types (ITs) data of F_3_ lines, the gene *YrBai* was further located in 0.8 cM region on wheat chromosome 6D. These closely linked markers developed in this study should be useful for MAS for Baidatou in crop improvement and map-based clone this gene.

## Introduction

Wheat (*Triticum aestivum* L.) is one of the most commonly grown cereal grain crops, but it is prone to three rust diseases: stripe rust, leaf rust and stem rust. Stripe rust, caused by *Puccinia striiformis* Westend. f. sp. *tritici* Eriks. (*Pst*), is a widely distributed and most devastating disease of wheat in world, such as Asia, Europe, Australia, North America, South America, the Middle East, and Africa (Wan et al., 2004; Chen, 2005; Wellings, 2011). This disease often results in 10–70% yield losses in susceptible cultivars (Li and Zeng, 2002; Chen, 2013). In China, especially the northwestern and southwestern regions including Gansu, Shaanxi, Qinghai, Ningxia, Sichuan, Yunnan, Guizhou, etc., stripe rust has caused significant economic losses during the last 60 years (Li and Zeng, 2002; Wan et al., 2007). Four nationwide stripe rust epidemics occurred in 1950, 1964, 1990, and 2002, which caused yield losses of 6.0, 3.2, 1.8, and 1.3 million metric tons or 29.3, 13.3, 1.8, and 1.4% of the national total production, respectively (Wan et al., 2004). The average area of disease occurrence is still more than 3.4 million hectares during 2003–2016 in China.

Fungicides have been applied widely to control wheat stripe rust, but they generate additional costs and are potentially harmful to the environment. Therefore, growing resistant cultivars is the most efficient, economically viable, and environment-friendly method to control this disease (Line and Chen, 1995; Chen, 2005). The stripe rust resistance mainly includes all-stage resistance (ASR) and adult-plant resistance (APR) (Qayoum and Line, 1985; Chen, 2005; Lin and Chen, 2007). ASR can be detected in the seedling stage and often provide high-level resistance, therefore, this type of resistance genes has been used widely in wheat breeding programs. However, due to race-specific nature and frequent virulence changes in pathogen populations, most of ASR genes lose the resistance they provide after 3–5 years of propagation, which is also the main reason for the four nationwide stripe rust epidemics in 1950, 1964, 1990, and 2002 in China. In contrast, APR is expressed during later stage of plant development, often effective to a broader range of races and confers more durable resistance (Lowe et al., 2011; Chen, 2013). To date, more than 70 officially named wheat stripe rust resistance genes and many temporarily designated genes or QTLs have been identified and mapped to specific wheat chromosomal locations (Dracatos et al., 2016; Li et al., 2016; Wu et al., 2016; Xiang et al., 2016; Dong et al., 2017). Unfortunately, most of these resistance genes have been not effective against current new *Pst* races. Therefore, ongoing genetic improvement for wheat stripe rust resistance requires the identification of more effective rust resistance genes.

Wheat landraces have unique characteristics including early maturity, high affinity to abiotic and biotic stresses, adaptability to kinds of ecological conditions, genetic diversity, heterogenicity, and grains, therefore, they have become one of the most important hereditary resources in wheat breeding programs. Moreover, wheat landraces provide a rich source of rust disease resistance genes to increase genetic diversity for stripe rust, leaf rust, and stem rust both pre-Green Revolution and now (Sthapit et al., 2014; Pasam et al., 2017). In China, wheat landraces were the main varieties before the 1960s (Zhou et al., 2017). Up to now, China has more than 13,000 wheat landraces accessions (Liu et al., 2000). The stripe rust resistance genes in Chinese wheat landraces Yilongtuomai and Hejiangzimai were located on wheat chromosome 7DS (Wu et al., 2016; Zhou et al., 2017). Three QTLs for APR for stripe rust, *QYr.caas-2BS, QYr.caas-5AL*, and *QYr.caas-6BS* were identified in Chinese wheat landrace Pingyuan 50 (Lan et al., 2010).

The Chinese wheat landrace Baidatou has been grown widely for several decades in Longnan region of Gansu Province, where stripe rust epidemics are frequent and severe. Although many *Pst* virulence changes have occurred in that region over the past decades, this variety is still highly resistant in the field. In our previous study (Ma et al., 2015), an APR gene *YrBai* was identified in Baidatou from two years field tests in Yangling, Shaanxi Province and located on wheat chromosome 6DS by four polymorphic simple sequence repeat (SSR) and two sequence-related amplified polymorphism (SRAP) markers, and the genetic distances of two closest flanking linked markers were 3.6 and 5.4 cM, respectively. The objectives of this study are: (1) to further confirm the APR gene in Baidatou; (2) to saturate the target region and construct the high-density genetic map for the APR gene.

## Materials and Methods

### Plant Materials

To investigate the inheritance and develop molecular markers for stripe rust resistance in Chinese wheat landrace Baidatou, the genotype was crossed with Mingxian 169. Mingxian 169, a winter wheat cultivar, is highly susceptible to all Chinese *Pst* races identified so far. Spikes on F_1_ plants were bagged prior to anthesis to prevent outcrossing. The F_2_ population was derived from a single F_1_ plant grown at the field during the 2011–2012 cropping season. F_2_ plants were planted in the field to obtain F_3_ lines during the 2012–2013 cropping season. 96 F_5:6_ recombinant inbred lines (RILs) were then developed by single-seed descent from 118 F_3_ lines.

The 118 F_3_ lines and 96 F_5:6_ RILs were used in Yangling field tests during 2014–2015, 2015–2016, and 2016–2017 cropping seasons, respectively. Also, 10 F_1_ seeds, 150 F_2_ seeds derived from the same single F_1_ cross spike as the 118 F_3_ lines were also used in the field tests during 2014–2015 crop season.

Wheat cultivars Fielder, Tres, and Lee, and F_2_ population derived from the cross Baidatou/Lee were tested in the field to determine if Baidatou and these cultivars share the same resistance gene on wheat chromosome 6D.

### Field Tests

Baidatou, Mingxian 169, and 118 F_3_ lines were evaluated for stripe rust reaction in the experimental fields at Yangling of Shaanxi Province during 2014–2015 and 2015–2016 crop seasons, respectively. Also, 96 F_5:6_ RILs and two parents were tested at the same sit in 2016–2017 crop season. The experiment was conducted in a randomized complete block design with three replications. Each replication comprised of one row of each parent and 118 rows of F_3_ lines or 96 rows of F_5:6_ RILs. About 20–30 seeds from each F_3_ or F_5:6_ line were planted in a 1 m row with 25 cM space between rows. About 10 F_1_ seeds and 150 F_2_ seeds were planted at Yangling site in 2014–2015 crop season. The trails were inoculated with the same predominant *Pst* race CYR33 as our previous study (Ma et al., 2015) at the beginning of stem extension stage in each year. Susceptible wheat cultivar Mingxian 169 was planted around each plot and after every 20 rows to increase the uniformity of infection throughout the field. The nurseries were managed using common practices for the regions.

Infection types (ITs) were recorded at the booting, heading-flowering and soft dough stages when rust severities on Mingxian 169 reached ∼30, 60, and 90%, respectively. ITs were recorded based on the 0–4 scale described by Bariana and McIntosh (1993). For the F_3_ lines and F_5:6_ RILs, ITs were recorded as a single value for homozygous lines and as two or more values for segregating lines.

### Phenotypic Data Analysis

F_1_, F_2_, F_3_, and F_5:6_ generations from the cross Mingxian 169/Baidatou were analyzed to determine the number of APR gene for stripe rust in Baidatou. The goodness of fit of observed numbers to expected frequencies for the phenotypic IT data was calculated with the “Chi-test” function in Microsoft Excel 2007.

### DNA Isolation

Genomic DNA was extracted from a random sub-set of 92 Mingxian 169/Baidatou F_3_ lines and both parents using a cetyltrimethyl ammonium bromide (CTAB) method (Saghai-Maroof et al., 1984). The DNA quality was determined by gel electrophoresis using a 1% agarose gel and spectrophotometry (NanoDrop ND-1000, Thermo Scientific, Wilmington, DE, United States). The concentration of DNA was normalized to 50 ng/μL.

### Genotyping Using DArTseq Analysis

Genotyping by sequencing analysis of two parents and 92 F_3_ lines were performed by Diversity Arrays Technology Pty Ltd (DArT P/L), Australia, as described by Raman et al. (2014) and Dracatos et al. (2016). DArTseq technology was optimized for wheat by selecting the most appropriate method for reducing genomic complexity and the *Pst*I-*Mse*I method was selected. DNA fragments were digested with restriction enzymes and ligated with *Pst*I adaptors and unique barcodes, then amplified following PCR. After PCR, equimolar amounts of amplification products from each sample of the 96-well microtiter plate were multiplexed and sequenced in a single lane on HiSeq2000 (Illumina, United States). Sequences generated from each lane were processed using proprietary DArT analytical pipelines. In the primary pipeline, the FASTQ files were first processed to filter away poor quality sequences; more stringent selection criteria ( ≥ Phred pass score of 30) were applied to the barcode region than to the rest of the sequence. As a result, the assignments of the sequences to specific samples carried in the barcode split step were very reliable. Approximately 2,000,000 sequences per barcode/sample were identified and used in marker calling. Finally, identical sequences were collapsed into FASTQ call files.

Based on DArTseq, two types of markers, SilicoDArT (presence and absence variations, also known as PAV markers) and SNP (single-nucleotide polymorphism), were generated. All the SilicoDArT and SNP markers were analyzed using DArTsoft v.7.4.7 (DArT P/L, Canberra, ACT, Australia). Several quality parameters, such as call rate (that is, the percentage of samples for which a given marker was scored), polymorphic information content (PIC), reproducibility (that is, the percentage of technical replicate pairs scoring identically for a given marker), and the average read depth (that is, the average number of sequence “tag” counts contributing to the genotype calls for a given marker), were automatically computed and used to filter both markers.

### Construction of Genetic Map

A total of 12,090 SilicoDArT markers and 7,813 SNP markers were obtained by DArTseq. Only the markers polymorphic between the resistant and susceptible parents were used for further analysis. The chromosomal locations of most of SilicoDArT and SNP markers have been provided by DArT P/L, Australia. However, some of SilicoDArT and SNP markers have been reported first time in this study and their chromosome location have not been described yet. Therefore, prior to map construction, markers were binned based on their segregation patterns in Mingxian 169/Baidatou F_3_ population using the Bin function in IciMapping V4.1 software (Wang et al., 2014). Chi-square goodness of fit test was conducted and markers that showed significant segregation distortion (*P* < 0.001) and miss data > 15% were removed. Then, the Map functionality of IciMapping V4.1 was used to group both SilicoDArT and SNP markers, with the previously mapped SilicoDArT and SNP markers (DArT P/L, Canberra, Australia) serving as anchored markers. A logarithm of the odds (LOD) score of 3.0 and a recombination fraction of 0.4 were used to sort the markers with the Kosambi mapping function (Kosambi, 1943). Groups were ordered with the Kosambi mapping function within the JoinMap 4.0 (Van Ooijen, 2006).

### Identification of Linkage Markers for *YrBai*

The genetic map information together with phenotyping data of F_3_ lines were used to identify the linkage markers for the stripe rust resistance gene *YrBai* using JoinMap 4.0 (Van Ooijen, 2006). Kosambi mapping function was used to convert recombination frequencies to genetic distances (Kosambi, 1943) and a LOD score of 3.0 was used as a threshold. The linkage map was graphically visualized with MapChart V2.3 (Voorrips, 2002).

### Genome Reference and Gene Annotation

To obtain physical positions of polymorphic SilicoDArT and SNP markers for APR gene *YrBai*, the sequence of these markers were blasted against the genome sequence of *T*. *aestivum* cv. Chinese Spring (Reference Sequence v1.0, RefSeq v1.0), the International Wheat Genome Consortium (IWGSC)^1^. Also, annotated genes in the target region were extracted from website^2^.

## Results

### Characterization of APR in Baidatou

**FIGURE 1:**
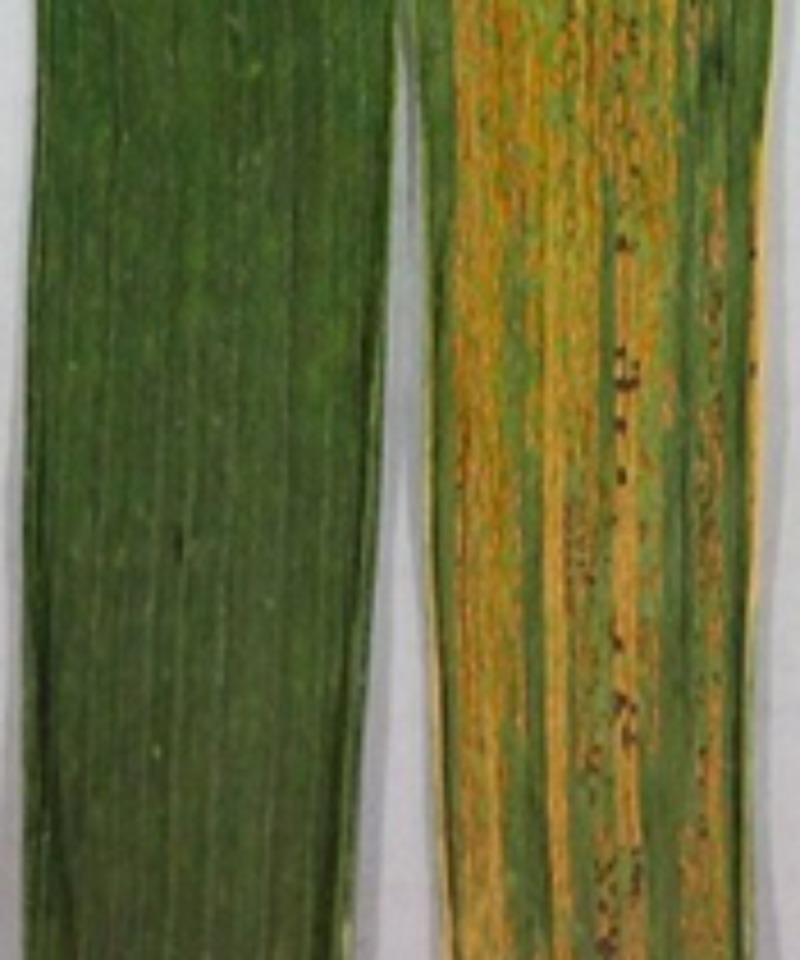
Adult plant leaves of Baidatou (left) and Mingxian 169 (right) inoculated with Chinese *Pst* race CYR33 at Yangling field in 2014–2015 crop season.

Baidatou exhibited high resistance (ITs 0–1 with DS 0–1%) (**Table 1** and **Figure 1**) at the adult-plant stage during all the tested crop seasons. Whereas Mingxian 169 was highly susceptible (ITs 3–4 with DS 80–100%). In 2014–2015 field trails (**Table 1**), all of the 10 F_1_ plants showed resistance. Of the 133 F_2_ plants, 107 plants were resistant and 26 plants were susceptible, fitting a 3 resistant:1 susceptible ratio (χ^2^= 2.11, *P* = 0.15). For 118 F_3_ lines, the number of homozygous resistant, segregating and homozygous susceptible lines was 27, 61, and 30, respectively, fitting 1 resistant : 2 segregating : 1 susceptible ratio (χ^2^= 0.29, *P* = 0.87). The same segregation ratio of F_3_ lines was obtained during 2015–2016 crop season (**Table 1**). Among 96 F_5:6_ RILs grown in 2016–2017 field trials were scored 56 homozygous resistant and 40 homozygous susceptible, fitting a single locus segregation ratio (χ^2^= 2.67, *P* = 0.10; **Table 1**). The above segregation data further confirmed that a single dominant gene *YrBai* is involved in APR to stripe rust in Baidatou.

**Table 1:** Genetic analysis of stripe rust resistance in progenies derived from the cross Mingxian 169/Baidatou at the adult plant stage in Yangling location during 2014–2015, 2015–2016, and 2016–2017 crop seasons.

Years	Parents and generations	No. of plants (lines)			Expected ratio	χ^2^	*P*
					
		Res.	Seg.	Sus.			
2014–2015	Baidatou	15		0			
	Mingxian169	0		15			
	F_1_	10		0			
	F_2_	107		26	3:1	2.11	0.15
	F_3_	27	61	30	1:2:1	0.29	0.87
2015–2016	Baidatou	14		0			
	Mingxian 169	0		15			
	F_3_	27	61	30	1:2:1	0.29	0.87
2016–2017	Baidatou	15		0			
	Mingxian 169	0		13			
	F_5:6_	56	–	40	1:1	2.67	0.10


*Res., Resistant; Seg., Segregated; Sus., Susceptible.*

### Construction of Genetic Map

**FIGURE 2:**
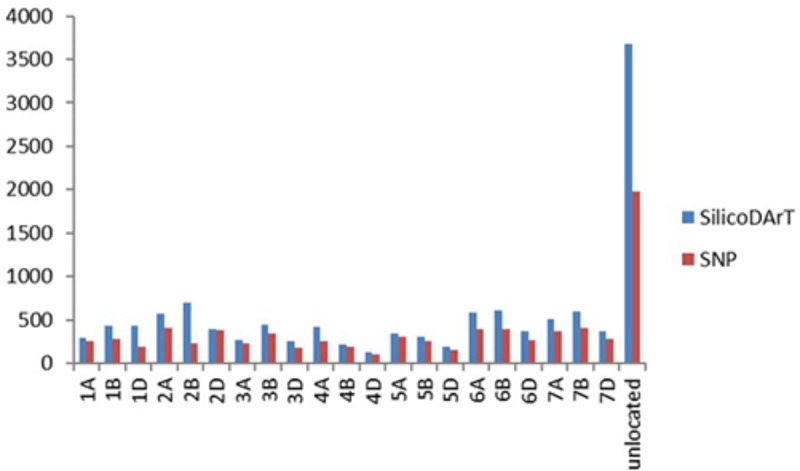
The distribution of SilicoDArT and SNP markers according to their chromosome location provided by DArT P/L, Australia.

A total of 12,090 SilicoDArT markers and 7,813 SNP markers were generated for 92 F_3_ lines of the cross Mingxian 169/Baidatou and two parents, which covered all 21 wheat chromosomes. The distribution of both SilicoDArT and SNP markers according to their chromosome location provided by DArT P/L, Australia is shown in **Figure 2**. The quality of these markers was assessed by different quality parameters, and the average call rate, PIC, reproducibility and read depth for each marker were 90.7%, 0.41, 0.98, and 8.45, ranging from 41 to 100%, 0.01 to 0.50, 0.90 to 1.00, and 1.51–137.11, respectively. The markers with Call rate > 85% and reproducibility equal to 1 were used in the further analysis.

Among these markers, 9,895 SilicoDArT markers and 3,029 SNP markers were polymorphic between the parents Baidatou and Mingxian 169. Of these polymorphic markers, 8,025 SilicoDArT markers and 2,731 SNP markers has been previously located on 21 wheat chromosomes by DArT P/L, Australia, respectively. After “Bin” and “Map” with IciMapping V4.1 software, 5,846 SilicoDArT and 2,865 SNP non-redundant markers including previously located and unlocated markers were all sort into 21 groups and used for genetic map construction.

The linkage map constructed for the F_3_ lines from the cross Mingxian 169/Baidatou comprised 31 linkage groups spanning 4,293.94 cM (**Table 2** and Supplementary Table 1) and the average distance was 1.39 cM. A total of 2,131 polymorphic SilicoDArT markers and 952 SNP markers were mapped in these 31 linkage groups. All 21 wheat chromosomes were represented across the 31 linkage groups. Most markers were mapped to the B (41.1%) and A genomes (39.3%), and only 19.6% markers were mapped on the D genomes.

**Table 2:** Summary of the linkage groups based on the F_3_ lines derived from the cross Mingxian169/Baidatou.

Linkage group	No. of SilicoDArT markers	No. of SNP markers	Total markers	Size (cM)	Mean distance (cM)
1A-1	87	13	100	143.59	1.44
1A-2	2	51	53	92.77	1.75
1B	122	12	134	279.78	2.08
1D	88	27	115	209.31	1.82
2A-1	34	5	39	85.25	2.18
2A-2	29	35	64	69.82	1.09
2B-1	35	50	85	96.44	1.13
2B-2	41	38	79	147.81	1.87
2D-1	55	4	59	121.49	2.06
2D-2	39	3	42	81.16	1.93
3A	98	54	152	183.16	1.21
3B-1	165	33	198	310.89	1.57
3B-2	123	96	219	256.88	1.17
3D	83	20	103	133.31	1.29
4A-1	115	19	134	165.79	1.24
4A-2	39	59	98	98.10	1.00
4B	51	36	87	113.15	1.30
4D	48	3	51	128.92	2.53
5A	89	30	119	140.43	1.18
5B	29	21	50	78.71	1.57
5D	17	1	18	55.73	3.10
6A-1	52	44	96	107.78	1.12
6A-2	42	44	86	133.99	1.56
6B-1	56	8	64	95.04	1.49
6B-2	58	46	104	138.63	1.33
6D	147	44	191	160.39	0.84
7A-1	153	11	164	262.14	1.60
7A-2	49	59	108	118.62	1.10
7B-1	123	7	130	92.34	0.71
7B-2	44	74	118	163.84	1.39
7D	18	5	23	28.68	1.25
Total	2131	952	3083	4293.94	–


### Identification of Linkage Markers for *YrBai*

**FIGURE 3:**
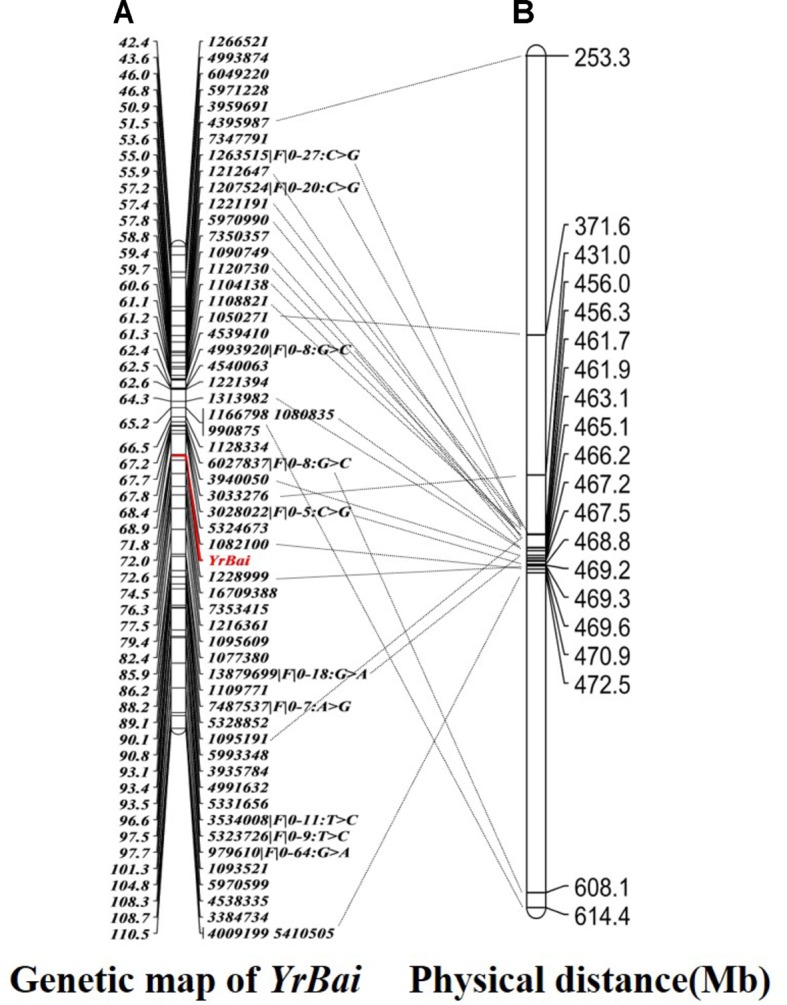
High-density genetic map **(A)** and physical position **(B)** of the adult-plant resistance gene *YrBai.*

Using JoinMap 4.0 software, 2131 polymorphic SilicoDArT markers, 952 SNP markers and field IT data of the cross Mingxian 169/Baidatou F_3_ lines were analysis to identify the linkage markers for the APR gene *YrBai*. The results indicated that 47 SilicoDArT markers and 10 SNP markers located on wheat chromosome 6D were linked to *YrBai*. The two closest flanking markers were SilicoDArT markers 1082100 and 1228999, which located *YrBai* on 0.8 cM region on wheat chromosome 6D (**Figure 3A**).

### Physical Map and Candidate Genes Analysis

The sequences of polymorphic SilicoDArT and SNP markers were blasted against the genome sequence of Chinese Spring (IWGSC RefSeq v1.0), most of markers were assigned to 16.6 Mb physical interval (6D:455988052-6D:472530090) (**Figure 3B**). According to gene annotation information in IWGSC RefSeq v1.0 databases, 360 predicted genes were obtained in this region (Supplementary Table 2). The two closest flanking markers 1082100 and 1228999 were all assigned to 470 Mb position (6D:470885770-6D:470908084). Therefore, 14 predicted genes related to disease resistance in this region maybe the candidate genes (**Table 3**). These genes included the typical nucleotide binding site-leucine-rich repeats (NBS-LRR) genes (*TraesCS6D01G400300*, *TraesCS6D01G400400*, and *TraesCS6D01G400700*), Receptor-like kinase (*TraesCS6D01G402600*), heat shock protein (*TraesCS6D01G402500*), etc.

**Table 3:** List of candidate genes in the closest flanking markers 1082100 and 1228999 and gene annotations based on Chinese Spring IWGSC RefSeq v1.0.

Gene ID	Hit-start	Hit-end	Human-readable-description
*TraesCS6D01G399900*	470046212	470047105	Transmembrane protein, putative (DUF594)
*TraesCS6D01G400000*	470047792	470048430	Transmembrane protein, putative (DUF594)
*TraesCS6D01G400100*	470065500	470066818	Cortactin-binding protein 2
*TraesCS6D01G400200*	470084602	470087613	Receptor kinase 2
*TraesCS6D01G400300*	470096737	470098901	NBS-LRR disease resistance protein
*TraesCS6D01G400400*	470098964	470100529	NBS-LRR-like resistance protein
*TraesCS6D01G400500*	470102303	470104546	Transmembrane protein, putative (DUF594)
*TraesCS6D01G400600*	470288675	470290934	Transmembrane protein, putative (DUF247)
*TraesCS6D01G400700*	470294277	470296952	NBS-LRR-like resistance protein
*TraesCS6D01G401000*	470543427	470544299	60 kDa chaperonin
*TraesCS6D01G401600*	470632933	470634909	Response regulator
*TraesCS6D01G402100*	470754620	470754898	Protein kinase family protein
*TraesCS6D01G402500*	470902402	470907928	70 kDa heat shock protein
*TraesCS6D01G402600*	470961431	470967108	Receptor-like kinase


### Relationship of *YrBai* to *Yr20, Yr23*, and *YrTr1* on Wheat Chromosome 6D

Wheat cultivars Fielder, Lee, and Tres, which carry stripe rust resistance genes *Yr20, Yr23*, and *YrTr1* located on wheat chromosome 6D, respectively, were tested with *Pst* race CYR33 in Yangling field during 2015–2016 crop season. Fielder and Tres were susceptible (ITs 3–4) and Lee was moderately resistant to moderately susceptible (ITs 2–3) to CYR33 at the adult-plant stage. However, Baidatou was highly resistant (ITs 0–1) to CYR33. Therefore, *Yr20* in Fielder and *YrTr1* in Tres were obvious different from *YrBai*.

To further determine the relationship between *Yr23* and *YrBai*, F_2_ population derived from the cross Lee/Baidatou was inoculated with *Pst* race CYR33 at the adult-plant stage in Yangling field during 2016–2017 crop season. The number of the resistant and susceptible F_2_ plants fit a 15 resistant:1 susceptible segregating ratio (χ^2^= 1.68, *P* = 0.20), suggesting that one dominant gene may come from Lee and another dominant gene maybe from Baidatou in the cross. Therefore, *YrBai* may be different from *Yr23*.

## Discussion

Stripe rust is one of the most important and destructive diseases of wheat in China. Due to the high frequent virulence changes of *Pst* races and loss of stripe rust resistance in most of widely used *Yr* genes, such as *Yr9, Yr3b*, and *Yr4b* (Wan et al., 2004; Chen et al., 2009), five to seven times large-scale replacements of commercial wheat cultivars have been carried out during the past several decades in China (Li and Zeng, 2002). However, Baidatou has kept high resistance to wheat stripe rust in Longnan region, Gansu Province since 1950s.

In our previous study (Ma et al., 2015), the adult-plants of F_1_, F_2_, and F_3_ populations derived from the cross Mingxian 169/Baidatou were inoculated with *Pst* race CYR33 in Yangling, Shaanxi Province, during 2009–2010 and 2010–2011 crop seasons, and the genetic analysis indicated that a single dominant gene (tentatively designated as *YrBai*) conferring APR in Baidatou. In this study, F_1_, F_2_, F_3_, and F_5:6_ generations derived from the same cross Mingxian 169/Baidatou were evaluated with the same *Pst* race CYR33 in Yangling field in 2014–2015, 2015–2016, and 2016–2017 crop seasons, respectively. The inheritance analysis of this study further confirmed that the APR in Baidatou was controlled by the single dominant gene *YrBai*. In addition, *YrBai* was located on wheat chromosome 6DS by four polymorphic SSR markers and two SRAP markers in our previous study, and the genetic distance of two flanking SSR markers was 3.6 and 5.4 cM, respectively (Ma et al., 2015). In the current study, *YrBai* was further located in 0.8 cM region on wheat chromosome 6D by polymorphic SilicoDArT and SNP markers. In the 14 candidate genes obtained from the two closest flanking markers region, wheat genes *TraesCS6D01G400300, TraesCS6D01G400400*, and *TraesCS6D01G400700* were NBS-LRR resistance genes and conferred high level resistance, which are the most similar as the resistance of *YrBai*. However, whether *YrBai* is one of the NBS-LRR resistance genes need further experiment to confirm.

To date, *Yr20, Yr23*, and *YrTr1* were located on wheat chromosome 6D by allelic test and monosomic analysis (Chen et al., 1995a,b). All of these genes showed ASR to wheat stripe rust, but *YrBai* in Baidatou showed APR. In addition, Fielder with *Yr20* and Tres with *YrTr1* were susceptible to *Pst* race CYR33 at the adult-plant stage, whereas, Baidatou was highly resistant. Lee with *Yr23* were moderately resistant to moderately susceptible to CYR33, moreover, the allelic test also indicated that *YrBai* might be different from *Yr23*. Therefore, *YrBai* should be different from *Yr20, Yr23*, and *YrTr1*, and maybe a novel APR gene.

Limited variation in elite germplasm may constrain deployment of diverse resistance genes in commercial wheat cultivars and the capacity for countering new virulence in pathogen populations (Peng et al., 2011). However, wheat landraces are mixtures of mostly homozygous genotypes that can tolerate to various abiotic and biotic stresses (Sthapit et al., 2014; Mangini et al., 2017). From 652 spring wheat landraces accessions collected from 54 countries, 165 accessions were identified to have resistance to wheat stripe rust, and 30 of the 165 accessions were also resistant to stem rust (Sthapit et al., 2014). For Baidatou in this study, it is highly resistant not only to wheat stripe rust but also powdery mildew (Cao et al., 2017). In addition to APR exhibited in most of wheat landraces, such as PI 480035 (Kandel et al., 2017) and Pingyuan 50 (Lan et al., 2010), some wheat landraces also show ASR to stripe rust, such as Yilongtuomai (Wu et al., 2016) and Laokao 5 (Yao et al., 2017). Kankwatsa et al. (2017) found that a range of possibly unidentified effective seedling and APRs present among wheat landraces, which might represent new sources of rust resistance. Feng et al. (2018) identified a novel gene *Yr79* and four additional QTLs for all-stage and high-temperature APR to stripe rust in wheat landrace PI 182103. Therefore, wheat landraces are very important sources to broaden the genetic base of cultivated wheat. Although possessing many disease resistance advantages, some wheat landraces have some poor agronomic traits or low yield potential. Therefore, the resistance gene closely linked markers are request in MAS to transfer desirable traits and exclude negative traits.

Most of APR show relatively small effects on stripe rust response with high IT and low severity and the resistances were controlled by several QTLs (Carter et al., 2009; Lan et al., 2010; Pawar et al., 2016; Li et al., 2017). However, Baidatou shows high resistance to stripe rust with IT 0–1 and the resistance was controlled by a single dominant gene, which made it more easily used in wheat breeding programs. These closely linked SilicoDArT and SNP markers developed in this study should be useful for MAS and promote the utilization of Baidatou in crop improvement.

In wheat improvement, resistance gene pyramiding not only enhances the efficacy and longevity of effective gene resistance against rust diseases (Singh et al., 2016), but also takes advantage of the weaker genes as well as those that have been partially overcome by current virulent pathotypes. In China, stripe rust resistance gene *Yr26* has been widely used in wheat breeding programs and varieties with *Yr26* have been grown over 3.4 million hectares in recent years, which resulted to occurrence and epidemic of *Yr26*-virulent races. The resistance of *Yr26* has been overcome again after *Yr9, Yr3b*, and *Yr4b* (Wan et al., 2004; Chen et al., 2009). Therefore, the stripe rust resistance gene shouldn’t be used alone in wheat breeding, especially for most of ASR genes. At the present, a few of ASR genes, such as *Yr5* and *Yr15*, and APR genes, such as *Yr18* and *YrZH22* (Wang et al., 2017), have kept effective resistance to Chinese predominant *Pst* races, including *Yr26*-virulent races. *YrBai* in Baidatou can be pyramid with these genes to obtain the durable resistant cultivars.

## Author Contributions

QL conducted the experiments, analyzed the data, and wrote the manuscript. JG, JY, WY participated in field experiments and contributed to the genotyping experiment. KC assisted in analyzing the data. DM participated in make the cross. BW conceived and directed the project and revised the manuscript.

## Conflict of Interest Statement

The authors declare that the research was conducted in the absence of any commercial or financial relationships that could be construed as a potential conflict of interest.

## Footnotes

1 https://urgi.versailles.inra.fr/blast_iwgsc/blast.php

2 https://wheat-urgi.versailles.inra.fr/Seq-Repository/Annotations
